# Political coherence and certainty as drivers of interpersonal liking over and above similarity

**DOI:** 10.1126/sciadv.abk1909

**Published:** 2022-02-09

**Authors:** Federico Zimmerman, Gerry Garbulsky, Dan Ariely, Mariano Sigman, Joaquin Navajas

**Affiliations:** 1 Laboratorio de Neurociencia, Universidad Torcuato Di Tella, Av. Figueroa Alcorta 7350, Buenos Aires C1428BCW, Argentina.; 2 National Scientific and Technical Research Council (CONICET), Godoy Cruz 2290, Buenos Aires C1425FQB, Argentina.; 3 Physics Department, Universidad de Buenos Aires, Av. Intendente Guiraldes 2160, Buenos Aires C1428EGA, Argentina.; 4 TED, Araoz 727, Buenos Aires C1414DPO, Argentina.; 5 The Fuqua School of Business, Duke University, 100 Fuqua Drive, Durham, NC 27708, USA.; 6 Facultad de Lenguas y Educación, Universidad Nebrija, Calle de Sta. Cruz de Marcenado 27, Madrid 28015, Spain.

## Abstract

Affective polarization and political segregation have become a serious threat to democratic societies. One standard explanation for these phenomena is that people like and prefer interacting with similar others. However, similarity may not be the only driver of interpersonal liking in the political domain, and other factors, yet to be uncovered, could play an important role. Here, we hypothesized that beyond the effect of similarity, people show greater preference for individuals with politically coherent and confident opinions. To test this idea, we performed two behavioral studies consisting of one-shot face-to-face pairwise interactions. We found that people with ambiguous or ambivalent views were nonreciprocally attracted to confident and coherent ingroups. A third experimental study confirmed that politically coherent and confident profiles are rated as more attractive than targets with ambiguous or ambivalent opinions. Overall, these findings unfold the key drivers of the affability between people who discuss politics.

## Teaser

Political coherence and certainty increase interpersonal liking above and beyond the effect of political homophily.

## INTRODUCTION

Over the past decades, the mutual dislike and distrust between people who hold different political opinions have been steadily increasing (*1*–*3*). Research has shown that this hostility spills over to nonpolitical domains, reducing cooperation in economic markets (*4*), hindering relationship with close family members (*5*), biasing the selection of romantic partners (*6*), and potentially contributing to the residential segregation of society (*7*). In this context of division and rising animosity between fellow citizens, understanding the key factors that predict interpersonal liking during political interactions has become a relevant and urgent issue in social science.

The standard explanation for the observed hatred between disagreeing individuals is that people show a preference for politically like-minded others (*8*, *9*), a phenomenon known as “political homophily” (*10*, *11*). The idea underlying this effect is simple: Two interacting individuals will like each other more if they share a larger proportion of opinions (*12*). Previous research has shown that the preference for similar people is prevalent in human social life and that it biases judgements across multiple domains. For example, people believe that politically like-minded individuals are better at solving cognitive tasks, even if those tasks have nothing to do with politics (*13*). This suggests that simply sharing the same political opinions with someone makes this person seem more knowledgeable and likeable.

However, while the liking-by-similarity effect stands as a largely accepted truth in psychological science (*14*), other factors could also play an important role, and assuming that liking is a univariate function of similarity may oversimplify the complexity of social interactions in the political domain. In this work, we hypothesized that, beyond the effect of similarity, interpersonal attraction depends on two other variables: coherence and certainty.

### Beyond political homophily

We say that someone is politically coherent if that person holds either all liberal or all conservative views across a wide range of issues. For example, an individual who supports same-sex marriage and is pro-choice on abortion (i.e., two traditionally liberal views) would be more politically coherent than a pro-life person who supports same-sex marriage (i.e., one conservative and one liberal view). Given a perceiver who interacts with a target, we define the target’s “ingroup coherence” as the number of opinions that are consistent with the perceiver’s ideology minus the number of ideologically inconsistent opinions. From the perspective of a liberal perceiver, ingroup coherence is computed as the number of liberal opinions held by the target minus the number of conservative opinions. Conversely, from the viewpoint of a conservative perceiver, the highest value of ingroup coherence is achieved when the target holds all conservative and no liberal opinions. This variable, which has also been called “ideological consistency” (*15*, *16*), takes positive values for pairwise interactions between political ingroups (e.g., two people with mostly liberal views) and negative values for interactions between political outgroups (e.g., one individual with mostly liberal views interacting with one holding mostly conservative views). We also define “attitude certainty” as the number of nonambiguous opinions held by the target. For example, an individual with well-defined liberal or conservative views on all issues would be more politically certain than someone who holds liberal or conservative opinions on some issues but is uncertain about their political views on some other topics.

While coherence and certainty are both related to the notion of political extremity, they are conceptually different variables (*17*–*19*). Extreme individuals tend to have ideologically consistent opinions (high coherence) and are confident about their views (high certainty). However, as previously noted (*20*), the reverse is not true: People with all liberal (or conservative) opinions might not necessarily be “extreme” as they could simply hold those views without being strong partisans or ever engaging in extreme political action. Likewise, moderate individuals might be so because they have low coherence (i.e., both liberal and conservative opinions), low certainty (i.e., some low-confidence opinions), or a combination of both. The aim of this work is to empirically disentangle the contributions of coherence and certainty to interpersonal attraction over and above the well-established effect of political similarity.

Our first hypothesis is that people show greater liking for individuals with higher ingroup coherence. Previous studies suggest that politically coherent individuals are more easily classified as prototypical ingroups or outgroups compared to those individuals having some degree of ambivalence in their attitudes (*21*–*24*). On the basis of previous findings demonstrating that people are attracted to pro-norm deviants (*25*, *26*), we reasoned that people should show greater liking for ingroups who are relatively more coherent than themselves. However, it remains unclear whether this hypothesized effect modulates interpersonal liking above and beyond the effect of homophily. We therefore empirically tested the hypothesis that, after accounting for the influence of similarity, people should feel most attracted to coherent ingroups and most repulsed by coherent outgroups.

The second hypothesis tested in this work is that people like individuals who are certain about their political opinions. Theoretical research has previously proposed that people’s identification with social groups is motivated by a need to reduce feelings of uncertainty about themselves and that interacting with confident others can help validate one’s own group affiliation (*27*, *28*). Moreover, a vast literature has shown that high-confidence individuals are perceived as more credible (*29*), persuasive (*30*), and influential (*31*, *32*) than people with low-confidence opinions. On the basis of these observations, we hypothesized that individuals with high political certainty are more likeable than people with uncertain views and test whether this attraction to confident others occurs at constant similarity and ingroup coherence.

### Empirical approach

Our goal is to empirically test whether political coherence and certainty modulate interpersonal liking above and beyond the well-known effect of similarity. Given that these three quantities—similarity, coherence, and certainty—are intermingled in most experimental settings, measuring the relative contribution of each variable to liking is a challenging endeavor that remains unaddressed. A natural approach to solve these intrinsic confounds is by experimentally dissociating these variables in artificial settings such as in the evaluation of phantom profiles (*33*). However, previous research has shown that these experimental manipulations usually do not extend to real interactions (*34*, *35*) and that they might artificially inflate the liking-by-similarity effect (*36*).

**Fig. 1.**
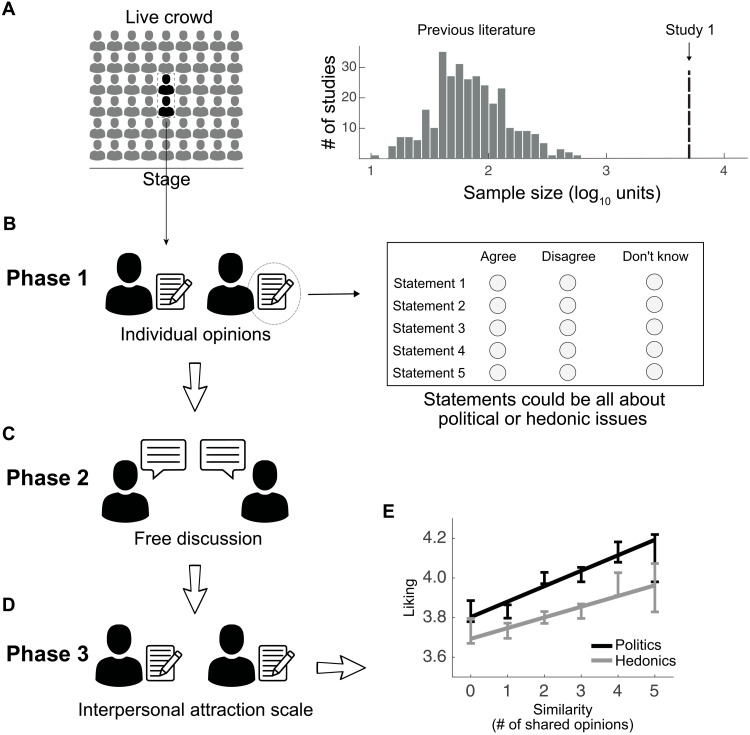
Experimental setup. (**A**) We performed a large-scale behavioral experiment with a live crowd. The right panel shows a histogram with the sample sizes of 310 previous studies measuring the liking-by-similarity effect, as reported in (*36*). The dashed line depicts the sample size of study 1 (*N* = 5038). (**B** to **D**) The experiment consisted of three phases. (B) Phase 1: Participants provided individual opinions about five statements by indicating whether they agreed or disagreed with each of them. They also had a third alternative to opt out in case they were uncertain about their opinion. Statements were all about political or hedonic issues. (C) Phase 2: Participants were organized into dyads and freely discussed their opinions for 5 min. (D) Phase 3: Participants completed a standard interpersonal attraction scale. (**E**) With this setup, we replicated the well-established correlation between liking and similarity for both the political and hedonic condition.

Here, we opted for a different and complementary approach: We capitalized on a recent program to run large-scale experiments with live crowds (*37*–*39*). This unique setting allowed us to collect data from thousands of individuals simultaneously performing a behavioral task. Previous editions of this program studied whether humans collaborate during a “zero-sum fallacy” game (*37*), the role of deliberation in the wisdom of crowds (*38*), and the factors that promote consensus in polarized moral issues (*39*). With this setup (Fig. 1A, right), we were able to increase sample size by two orders of magnitude compared to typical laboratory settings and collect data from a large number of participants (Fig. 1B). In this way, we could observe infrequent but naturally occurring dissociations between similarity, coherence, and certainty while still measuring interpersonal liking during real face-to-face pairwise interactions.

We performed our first study (study 1, *N* = 5038) in Argentina, a country with long-standing political polarization both at the ideological (*40*) and affective (*41*) level. To test the replicability and external validity of our observations, we performed a second independent study in Portugal (study 2, *N* = 838), a highly polarized country (*42*). Last, we supplement these observational studies with an experiment, performed in the United States (study 3, *N* = 400), where we directly manipulated the coherence and certainty expressed in the target’s political statements and study the liking ratings induced by this manipulation. To anticipate our findings, all studies provided evidence supporting the hypotheses that political coherence and certainty modulated interpersonal liking above and beyond the effect of political similarity.

## RESULTS

### Testing the effect of political similarity on liking

We performed a classic behavioral experiment in a live crowd (Fig. 1) and collected data from a large number of pairwise face-to-face interactions (study 1: *N* = 5038; Fig. 1A, right). The experimental design consisted of three phases (see “Experimental procedure” in Materials and Methods for details). In the first phase (Fig. 1B), we asked participants to read five statements and write down whether they agreed or disagreed with each of them. Participants could also choose a third option to opt out if they felt uncertain about their opinion (Fig. 1B, right). Approximately half of the participants (*N* = 2632) responded to five political issues (e.g., “high school students should be allowed to go on strike”), while the remaining others (*N* = 2406) responded to hedonic statements (e.g., “fried schnitzels taste better than baked schnitzels”). While the two categories of statements are evidently different (see the “Selection of the statements” section in Materials and Methods), previous research had shown that political divides sometimes extend to everyday likes (*43*). In accordance with this finding, we observed that both types of statements elicited similar levels of disagreement in the crowd [i.e., the fraction of participants who selected “agree,” averaged across statements, was 43.0% for politics and 43.3% for hedonic issues; chi-square test for proportions, χ^2^(1) = 0.14, *P* = 0.71; fig. S1].

In the second phase (Fig. 1C), participants were organized in dyads and freely discussed for 5 min their opinions about the five statements that they had just read. Dyads were always formed by two participants receiving the same set of statements. The debates were completely free and unstructured, but to make sure that all opinions were interchanged, we requested participants to write down the other person’s answers in their own sheet (see Materials and Methods for details). Last, in the third phase (Fig. 1D), participants rated their partner in terms of whether they seemed nice, charming, intelligent, candid, and physically attractive. As expected from the literature (*44*, *45*), these five ratings were significantly correlated with each other (Spearman correlation, *r* > 0.19, *P* < 4 × 10^−40^).

To ensure that dyads were formed mostly by unacquainted individuals, we organized them across different rows (Fig. 1, A and B). Since people tend to sit in a theater next to other people they know, this manipulation led to a large proportion of interactions between people who did not know each other before the experiment (88.0%). We therefore set a strict criterion for data exclusion and discarded all data proceeding from participants who were acquainted to each other. We also confirmed that the matching between participants across consecutive rows was consistent with a random procedure, given the observed distribution of shared opinions (see fig. S2 and Materials and Methods for details).

As expected from the literature (*12*), we found that liking increased as a function of similarity (Fig. 1E) and that this effect was present for both political and hedonic issues [linear mixed model; see Materials and Methods for details; politics: β = 0.11 [0.07, 0.15], SE = 0.02, *t*(2630) = 5.4, *P* = 9 × 10^−8^, *R*^2^ = 0.04; hedonics: β = 0.07 [0.03, 0.11], SE = 0.02, *t*(2404) = 3.4, *P* = 8 × 10^−4^, *R*^2^ = 0.07]. We also observed, however, two differences between the political and hedonic conditions. First, the fraction of variance explained by similarity was larger in the hedonic domain (politics: *R*^2^ = 0.04; hedonics: *R*^2^ = 0.07), suggesting that the liking-by-similarity model provides better fits to liking ratings in the hedonic condition compared to the political condition. Second, we found that liking ratings were generally higher in people who discussed political issues compared to those who discussed hedonic issues [politics: *M* = 3.96, SE = 0.02; hedonics: *M* = 3.80, SE = 0.02; two-sample *t* test, *t*(5036) = 6.4, *P* = 2 × 10^−10^]. This latter finding is consistent with the idea, previously reported in the literature (*46*, *47*), that the attraction between individuals depends on the category and importance of the discussed items. We did not find any evidence that the effect of similarity was more pronounced in one condition or the other [interaction between similarity and a dummy variable coding for “politics,” β = 0.03 [−0.02, 0.09], SE = 0.03, *t*(5035) = 1.1, *P* = 0.25].

### Testing the effects of political coherence and certainty on liking

**Fig. 2.**
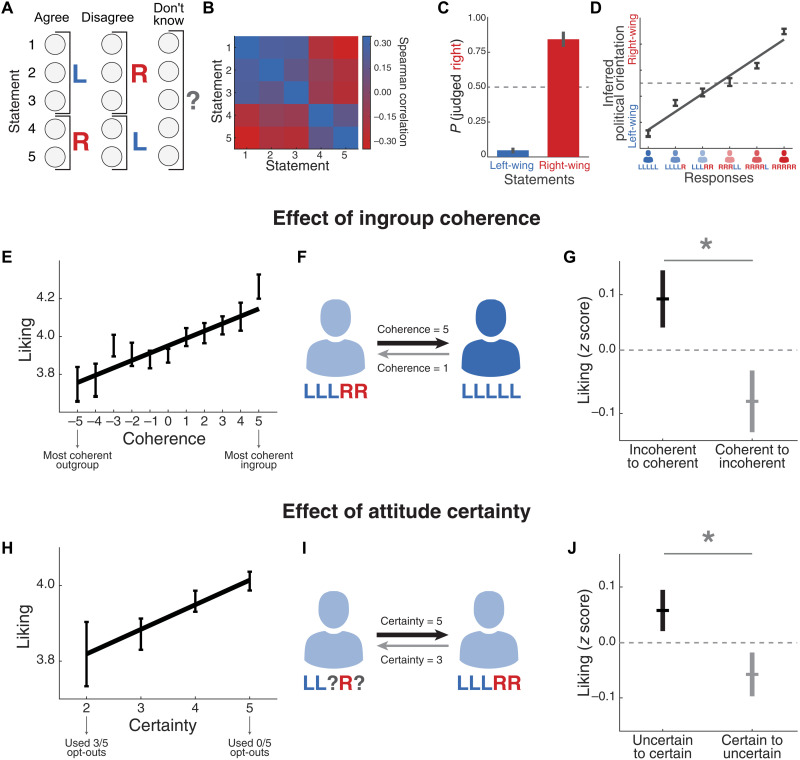
Analysis of interactions between people who discussed political statements. (**A**) In the political condition of study 1, three of the five statements were framed as a left-wing opinion and the remaining two as a right-wing opinion. (**B**) Colors code the Spearman correlation coefficients between all pairs of statements based on participants’ agreement responses. (**C**) To ensure that the statements that we defined as endorsing a “left-wing” or “right-wing” opinion were actually perceived in that manner, we asked participants to classify them in a separate control study (*N* = 35). The height of the bars depicts the probability that a participant judged a given statement as right-wing, and vertical lines show the SE of proportion. (**D**) In the same control study, participants were shown six profiles of responses and were asked to infer their political orientation. The inferred political orientation correlated with the balance of left- versus right-wing opinions endorsed in the profiles. (**E**) In study 1, we observed that interpersonal liking increased as a function of ingroup coherence. (**F**) We selected dyads with different degrees of coherence in their opinions and asked whether liking ratings were non-reciprocal (*n* = 345 dyads). (**G**) Within those dyads, liking ratings provided by the less-coherent participants were higher than the ones provided by the relatively more-coherent individuals. Dots depict mean *z*-scored liking ratings, and bars show SEM (*P < 0.05). (**H**) Liking ratings increased as a function of attitude certainty. (**I**) We selected dyads with different degrees of certainty in their opinions to study whether liking ratings were nonreciprocal (*n* = 678 dyads). (**J**) Within those dyads, ratings provided by the less-certain participants were higher than the ones provided by the relatively more-certain individuals. Dots depict mean *z*-scored liking, and bars show SEM (*P < 0.05).

After replicating the well-established correlation between liking and similarity, we then addressed the key question of this study: Could ingroup coherence and certainty also modulate interpersonal liking? To answer this question, we focused on the data obtained from dyads who discussed political issues. Political statements were framed in a way such that three of them reflected a left-wing opinion and two of them a right-wing opinion (Fig. 2A). A principal components analysis (PCA) applied on participants’ responses revealed that only one dimension explained a significant proportion of the variance in the data (37.0% of the variance, chance level: 20%, random permutation test, *P* < 1 × 10^−4^) and that this dimension was consistent with the left- versus right-wing nature of the statements (Fig. 2B). In an independent control study (see Materials and Methods for details), we confirmed that these phrases were indeed perceived as reflecting a stereotypical left- or right-wing opinion [i.e., “L” or “R,” respectively; chi-square tests for proportions, χ^2^(1) > 17.1, *P* < 3 × 10^−5^; Fig. 2C]. We also observed that people inferred the political orientation of other individuals based on the balance between the number of left- and right-wing opinions that they endorsed [control study, β = 0.84 [0.77, 0.91], SE = 0.04, *t*(208) = 22.4, *P* = 3 × 10^−57^, *R*^2^ = 0.7; Fig. 2D].

We quantified ingroup coherence (Fig. 2E) as the number of opinions that are consistent with one’s own political leaning minus the ones that are consistent with the opposite political orientation (for more details, see Materials and Methods). In accordance with our first hypothesis, we found that interpersonal liking increased as a function of ingroup coherence [β = 0.11 [0.08, 0.15], SE = 0.02, *t*(2630) = 5.7, *P* = 1 × 10^−8^, *R*^2^ = 0.04; Fig. 2E]. One limitation of this analysis is that simply showing a correlation between coherence and liking does not inform us about whether this modulation occurs at constant similarity. To tackle this issue, we focused on a key property of ingroup coherence: its asymmetry. If liking is solely driven by similarity, then ratings should be approximately reciprocal given its symmetric nature. However, if coherence also plays a role at constant similarity, then ratings should be nonreciprocal, and we should observe that ambivalent individuals show greater liking for coherent ingroups than vice versa (Fig. 2F). Our observations provided evidence for the latter idea (Cohen’s *d* = 0.14, Wilcoxon test, *z* = 2.7, *P* = 0.007; Fig. 2G).

We then tested the second hypothesis of this work that attitude certainty predicts interpersonal liking. Following a vast literature using “opt-out paradigms” (*48*), we defined a variable called “certainty” as the number of opinions where participants did not choose the opt-out option (see Materials and Methods for details). We found that liking increased as a function of certainty [β = 0.07 [0.03, 0.11], SE = 0.02, *t*(2630) = 3.6, *P* = 3 × 10^−4^, *R*^2^ = 0.04; Fig. 2H], and so we asked whether this effect could still be present at constant similarity. To answer this question, we selected dyads where one participant had used the opt-out option more times than the other (Fig. 2I). We found that, within these interactions, the relatively more-uncertain individuals provided higher liking ratings to the more-certain participants than vice versa (Cohen’s *d* = 0.08, Wilcoxon test, *z* = 2.2, *P* = 0.03; Fig. 2J). This suggests that interactions were nonreciprocal and that certainty modulated liking above and beyond the symmetric effect of similarity.

To formally measure the relative contribution of similarity, coherence, and certainty, we fitted a multivariate mixed-effects linear regression model on liking ratings (Table 1; see Materials and Methods for details). Our results indicate that the effect of coherence [β = 0.10 [0.05, 0.14], SE = 0.02, *t*(2628) = 3.9, *P* = 1 × 10^−4^] and certainty [β = 0.06 [0.02, 0.10], SE = 0.02, *t*(2628) = 2.8, *P* = 0.006] remained significant after controlling for similarity [β = 0.03 [−0.02, 0.09], SE = 0.03, *t*(2628) = 1.3, *P* = 0.19]. We also found that the proposed model (i.e., liking as a function of similarity, coherence, and certainty) provided better fits to the data than a model based solely on similarity, as diagnosed by the Akaike Information Criterion (δAIC = 14.7). Consistent with this observation, a likelihood-ratio test determined that the extended model with two additional free parameters is justified [χ^2^(2) = 18.7, *P* = 9 × 10^−5^]. In addition, we observed that the effects of coherence and certainty were robust to the inclusion of demographic control variables [effect of age: β = −0.06 [−0.01, −0.02], SE = 0.02, *t*(2053) = −2.8, *P* = 0.005; effect of gender: β = −0.04 [−0.08, 0.01], SE = 0.02, *t*(2053) = −1.8, *P*= 0.08].

**Table 1.** Formal model comparison. Comparison between the full multivariate mixed-effects model of liking as a function of similarity, coherence, and certainty and the restricted univariate model, where liking is a function of only similarity (i.e., coherence and certainty estimates are constrained to 0). Columns show the standardized coefficient estimates ± SEM, *t* values, and *P* values of each predictor. For each model, rows depict similarity (Eq. 5), coherence (Eq. 6), certainty (Eq. 4), log-likelihood, and the AIC. In the last row, we display the statistics of the likelihood-ratio test, which shows that the full model provides a significantly better fit to the observed data.

**Study 1**	**Restricted model**			**Full model**		
	**β**	***t***	***P***	**β**	***t***	***P***
Similarity	0.108 ± 0.020	5.37	9 × 10^−8^	0.034 ± 0.026	1.31	0.19
Coherence	–			0.095 ± 0.024	3.90	1 × 10^−4^
Certainty	–			0.058 ± 0.021	2.76	0.006
Log-likelihood	−3713			−3703		
AIC	7434			7419		
Likelihood-ratio test	χ^2^(2) = 18.7, *P* = 9 × 10^−5^					

One limitation of the nonreciprocities observed in Fig. 2 (G and J) is that they cannot disentangle whether the effects are driven by qualities of the perceiver or the target. To address this issue, we looked at dyads where the perceiver had a constant level of coherence or certainty and studied whether liking ratings were still modulated by the target’s coherence or certainty while accounting for the influence of similarity. Conditioning the data on dyads where the perceivers had a level of coherence of three (e.g., someone with four liberal and one conservative opinions; *N* = 511), we found that liking increased with the target’s coherence after accounting for the influence of similarity [β = 0.16 [0.04, 0.27], SE = 0.06, *t*(508) = 2.6, *P* = 0.01]. Critically, if we look at liking within a level of coherence of three in the target and study whether the perceiver’s coherence modulates liking above similarity, we do not find evidence for such an effect [β = 0.01 [−0.11, 0.13], SE = 0.06, *t*(523) = 0.21, *P* = 0.84].

Similarly, when we repeated this analysis conditioning the data on dyads where the perceivers had a value of certainty equal to three (e.g., someone with three liberal and two “do not know” answers, *N* = 464), we found that liking increased with the target’s certainty after accounting for the influence of similarity [β = 0.097 [0.003, 0.191], SE = 0.048, *t*(461) = 2.0, *P* = 0.04]. The opposite procedure, where we left constant the target’s certainty, showed that liking did not increase with the perceiver’s certainty [β = −0.02 [−0.11, 0.08], SE = 0.05, *t*(467) = −0.35, *P* = 0.73]. In line with these results, we observed (using the entire dataset) that the perceivers’ coherence and certainty did not significantly modulate interpersonal liking after accounting for the influence of similarity and the target’s coherence and certainty [perceiver’s coherence: β = 0.01 [−0.05, 0.07], SE = 0.03, *t*(2626) = 0.42, *P* = 0.67; perceiver’s certainty: β = −0.03 [−0.08, 0.01], SE = 0.02, *t*(2626) = −1.6, *P* = 0.12]. These findings suggest that if the perceiver’s coherence and certainty played a role in our data, their influence is negligible compared to the effect given by those same qualities in the target’s opinions.

Next, we fitted the proposed model to each individual item in the interpersonal attraction scale to study which aspects of liking were more influenced by each variable. We found that the main results were strongly present in the two items where participants rated the charm and intelligence of their partner. The remaining three individual items (niceness, candidness, and physical attractiveness) were not significantly modulated by either similarity, coherence, or certainty (fig. S3).

Last, we studied whether the effects of coherence and certainty were selective to the political domain. For this analysis, we focused on participants discussing hedonic issues (*N* = 2406; Fig. 1B) and projected the data onto the first principal component, which explained a significant proportion of the variance (27.3%, permutation test, *P* < 1 × 10^−4^). Once we defined these stereotypical responses, we were able to define coherence, certainty, and similarity in exactly the same way as we defined those variables in the political domain (see Materials and Methods for mathematical definitions). In this case, the multivariate model showed that coherence [β = 0.01 [−0.04, 0.06], SE = 0.02, *t*(2402) = 0.46, *P* = 0.65] and certainty [β = 0.01 [−0.03, 0.05], SE = 0.02, *t*(2402) = 0.46, *P* = 0.65] did not modulate liking and that only the effect of similarity remained significant [β = 0.06 [0.01, 0.11], SE = 0.03, *t*(2402) = 2.5, *P* = 0.01]. We also observed that the restricted model with one parameter provided better fits than the extended multivariate model [log-likelihood ratio test: χ^2^(2) = 0.36, *P* = 0.83]. This analysis suggests that the effects of coherence and certainty may be specific to political interactions.

### Replication in a different country

**Fig. 3.**
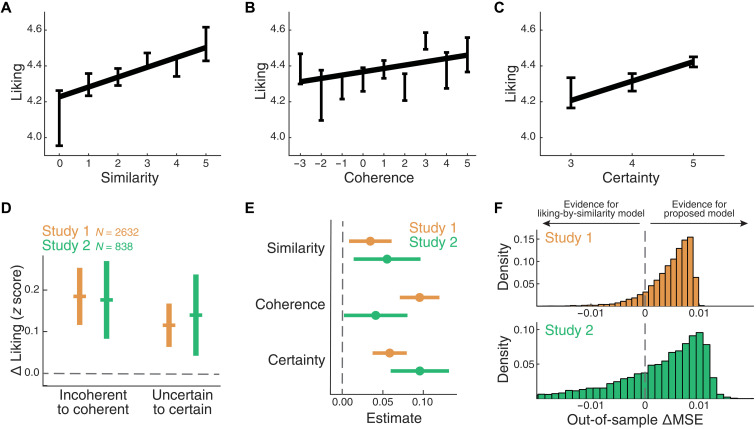
Empirical replication in a different country and language. (**A** to **C**) As in study 1 (Argentina), liking ratings obtained in study 2 (Portugal) correlated with similarity (A), coherence (B), and certainty (C). Error bars show means ± SEM, and we display the best-fitting line. (**D**) In study 1 (*N* = 2632 individuals who discussed political statements) and study 2 (*N* = 838), we observed evidence that liking was nonreciprocal among dyads with different levels of coherence and certainty. Orange bars show the difference in liking given by these two effects in study 1, and green bars show the same for study 2. Vertical lines show SEM. (**E**) The estimates of the multivariate mixed model of liking as a function of similarity, coherence, and certainty are shown in orange for study 1 and green for study 2. Vertical lines show SEM. (**F**) We measured the out-of-sample accuracy of the full mixed model versus the restricted liking-by-similarity model. Histograms show the difference in out-of-sample MSE (ΔMSE) of both models, with negative values indicating evidence in favor of the proposed model. The height of the bars reflects probability density across 10,000 simulations with different random splits of the data between equally sized training and testing sets (see Materials and Methods for details).

We sought to replicate our findings in a second study that took place in a different country and language (study 2, *N* = 838). As in study 1, we found that liking correlated with similarity [β = 0.10 [0.03, 0.17], SE = 0.04, *t*(836) = 2.8, *P* = 0.005, *R*^2^ = 0.05; Fig. 3A], coherence [β = 0.07 [−0.01, 0.13], SE = 0.04, *t*(836) = 1.8, *P* = 0.07, *R*^2^ = 0.04; Fig. 3B], and certainty [β = 0.11 [0.04, 0.18], SE = 0.03, *t*(836) = 3.2, *P* = 0.001, *R*^2^ = 0.05; Fig. 3C]. We also found that the two non-reciprocal effects found in study 1 (Fig. 2, F and I) were equally strongly present in study 2 (permutation test, coherence: *P* = 0.02, certainty: *P* = 0.02; Fig. 3D), including the observation that the effect of certainty (Cohen’s *d* = 0.10) was smaller than the effect of coherence (Cohen’s *d* = 0.14).

When we fitted the proposed model to the data obtained in study 2 (table S1), we found that the best-fitting coefficients overlapped with the ones observed in study 1 [similarity: β = 0.06 [−0.03, 0.14], SE = 0.04, *t*(834) = 1.3, *P* = 0.18; coherence: β = 0.04 [−0.04, 0.12], SE = 0.04, *t*(834) = 1.0, *P* = 0.30; certainty: β = 0.10 [0.02, 0.17], SE = 0.04, *t*(834) = 2.7, *P* = 0.008; Fig. 3E]. In accordance with this result, the AIC of the trivariate model was lower than the one based solely on similarity [δAIC = 3.5, log-likelihood ratio test: χ^2^(2) = 7.5, *P* = 0.02]. Last, we performed a quantitative model comparison analysis using out-of-sample testing and found that the restricted model led to poorer cross-validated performance compared to the unrestricted model based on similarity, coherence, and certainty. This was observed both in study 1 [paired *t* test on out-of-sample mean squared error (MSE), *t*(9999) = 124.0, *P* < 1 × 10^−300^; paired *t* test on out-of-sample negative log-likelihood, *t*(9999) = 123.8, *P* < 1 × 10^−300^; Fig. 3F, top] and in study 2 [paired *t* test on out-of-sample MSE, *t*(9999) = 19.7, *P* = 5 × 10^−85^; paired *t* test on out-of-sample negative log-likelihood, *t*(9999) = 20.1, *P* = 4 × 10^−88^; Fig. 3F, bottom]. This empirical replication suggests that our results were not driven by the country-specific political context associated with study 1.

### The interplay between similarity, coherence, certainty, and liking

Both studies showed that the effect of similarity on liking became smaller, even nonsignificant, when coherence and certainty were added to the model. To test for a potential model specification problem, we first studied whether multicollinearity played an important role in the data. To address this concern, we looked at the *R*^2^ value of a regression of similarity on coherence and certainty. We observed, however, no indication of severe multicollinearity in our data as the variance inflation factor (VIF) was well below standard thresholds used in the literature (regression of similarity on coherence and certainty, study 1: *R*^2^ = 0.44, VIF = 1.77; study 2: *R*^2^ = 0.27, VIF = 1.38) (*49*).

**Fig. 4.**
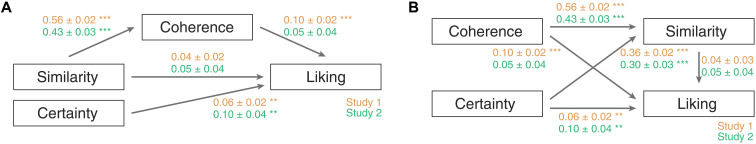
Interplay between similarity, coherence, certainty, and liking. (**A**) Mediation (MED) model. We fitted a model where the effect of similarity on liking is mediated by coherence while certainty directly predicts liking. This model provided better fits than a model where certainty is the mediator. (**B**) Confounding variable (CV) model. We fitted another model where similarity and liking are both driven by coherence and certainty. This account would suggest that the liking-by-similarity effect is confounded by the influence of coherence and certainty on both variables. In both panels, we display the best-fitting coefficients ± SE, and asterisks indicate the significance level of each estimate (**P < 0.01, and ***P < 0.001). Orange, study 1; green, study 2. Our observations are more likely under the CV model than under the MED model.

Another possibility is that the effect of similarity on liking was reduced because it was mediated by coherence or certainty. For example, if people who discussed with similar others were more likely to debate with coherent ingroups, then similarity would have increased coherence, and coherence might have influenced similarity. In line with this idea, we found a significant reduction in the direct effect of similarity after including coherence as a mediator, although this effect was only significant in study 1 (total effect = 0.09 [0.05, 0.13], *P* < 2 × 10^−16^; direct effect = 0.04 [−0.01, 0.08], *P* = 0.15; indirect effect = 0.05 [0.03, 0.08], *P* < 2 × 10^−16^) but not in study 2 (total effect = 0.07 [0.00, 0.15], *P* = 0.07; direct effect = 0.05 [−0.03, 0.13], *P* = 0.24; indirect effect = 0.02 [−0.01, 0.05], *P* = 0.28). This mediation model, where coherence but not certainty mediates the liking-by-similarity effect (Fig. 4A), provided better fits to the data than a model where certainty is the mediator of similarity (study 1: δAIC = 620; study 2: δAIC = 91).

Yet another alternative is that, in our studies, people with more coherent and certain opinions were more likely to interact with similar others. In that model, coherence and certainty would influence both similarity and liking, and the liking-by-similarity effect (Fig. 1E) would have been confounded by the effects of coherence and certainty on both variables (Fig. 4B). In line with this idea, a structural equation model suggested that this confounding variable model, where coherence and certainty modulate both similarity and liking, provides better fits to the data than the previously described mediation model (study 1: δAIC = 529; study 2: δAIC = 96). This result suggests that the observed correlation between liking and similarity (Fig. 1E) could have been mostly driven by the influence of two confounding variables: ingroup coherence and certainty.

However, one main limitation in the abovementioned conclusion is that the results presented so far rely on observational data, which cannot rule out the possibility that a latent variable drove all these effects. For example, given previous research showing that acquiescent people are more likely to report not knowing about political matters (*50*–*52*), we might be capturing the effect of acquiescence (which may causally affect certainty) on liking. To address this “third variable” problem, we performed a new study.

### Experimental study

We performed a third study where we experimentally manipulated the target’s coherence and certainty while measuring interpersonal attraction and participants’ perceptions of the targets’ similarity, coherence, and certainty (study 3). Our sample included *N* = 400 U.S. citizens recruited through Prolific (https://prolific.co/; see Materials and Methods for details) and was balanced in terms of ideology (liberal/conservative) and gender (male/female).

**Fig. 5.**
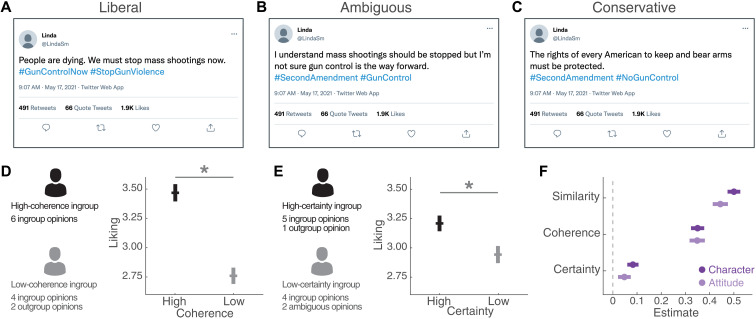
Experimental manipulation of coherence and certainty. (**A** to **C**) We performed a study with participants from the United States (*N* = 400), where they rated two putative Twitter users, each one expressing opinions on six different issues: immigration, global warming, police brutality, same-sex marriage, gun control, and COVID-19 vaccines. Tweets were written either as supporting a liberal opinion (A) (supporting gun control legislation), an ambiguous opinion (B) (uncertain about whether gun control should be enforced), or a conservative one (C) (opposing gun control legislation). (**D** and **E**) Participants were pseudo-randomly assigned to two between-subjects conditions: In one condition, they rated profiles with different levels of ingroup coherence at constant certainty, and in the other condition, the targets had different levels of certainty at constant ingroup coherence. (D) In the first condition, the high-coherence target was rated more positively than the low-coherence one. (E) In the second condition, the high-certainty target elicited higher liking ratings than the low-certainty profile. (**F**) Participants also rated the profiles in terms of whether they believed that they were coherent, certain, and similar to themselves. They did so by rating their perceived similarity/coherence/certainty in their attitudes and in their character. Error bars display the best-fitting coefficients (± SE) of perceived similarity, coherence, and certainty on liking. Dark purple, perceived character similarity/coherence/certainty; light purple, perceived attitude similarity/coherence/certainty.

Participants were presented with two putative Twitter users, each expressing six political opinions in different tweets (Fig. 5). Opinions were about six different issues: immigration, global warming, police brutality, same-sex marriage, gun control, and coronavirus disease 2019 (COVID-19) vaccines (for a full description of each expressed opinion, see Materials and Methods). Tweets were framed in a way in which they could either express a liberal opinion (Fig. 5A), an ambiguous opinion (Fig. 5B), or a conservative one (Fig. 5C). The presentation order of the two profiles, as well as their six opinions, was randomly chosen for each participant. Immediately after reading the six opinions of each user, participants rated whether they believed that such person was similar to themselves as well as their political coherence and certainty. Last, they completed a standard interpersonal attraction scale (*44*, *53*, *54*).

Participants were randomly assigned to two conditions in a between-subjects design. In the first condition, they rated two ingroup targets with different degrees of coherence in their attitudes. For example, liberal participants were presented with one target holding six liberal opinions (“high coherence”), while the other target had four liberal and two conservative opinions (“low coherence”). Conversely, for conservative participants, the high-coherence target had six conservative opinions, and the low-coherence target had four conservative and two liberal opinions. In the second condition, participants rated two ingroup targets who had different degrees of certainty in their attitudes. For example, liberal participants rated one target with five liberal opinions and one conservative opinion (“high certainty”), while the other target had four liberal and two ambiguous opinions (“low certainty”). For conservative participants, the high-certainty target held one liberal and five conservative opinions, and the low-certainty target had four conservative and two ambiguous opinions. This experimental design, hypotheses, and planned analyses were preregistered at https://aspredicted.org/q7kr6.pdf.

First, we tested whether the experimental manipulation of the target’s opinions elicited different ratings of interpersonal attraction. We found that the high-coherence profile was rated as more attractive than the low-coherence profile (high coherence: *M* = 3.47, SE = 0.07; low coherence: *M* = 2.76, SE = 0.07; Wilcoxon signed-rank test, *z* = 7.3, *P* = 3 × 10^−13^; Fig. 5D). We also observed higher liking ratings elicited by the high-certainty target compared to the low-certainty target (high certainty: *M* = 3.21, SE = 0.07; low certainty: *M* = 2.94, SE = 0.07; *z* = 3.2, *P* = 0.002; Fig. 5E).

The second line of planned analyses involved evaluating whether subjective perceptions about the target’s coherence and certainty increased interpersonal attraction after accounting for the influence of perceived similarity. We measured participants’ perceptions by asking whether they believed that the targets were similar, coherent, and certain in character (“perceived character similarity, coherence, or certainty”) and in their attitudes (“perceived attitude similarity, coherence, or certainty”). For both metrics, we found that a model including similarity, coherence, and certainty (Fig. 5F) provided better fits to liking ratings than a restricted model based only on perceived similarity [character: δAIC = 205, χ^2^(2) = 209, *P* < 1 × 10^−300^; attitude: δAIC = 136, χ^2^(2) = 140, *P* < 1 × 10^−300^; see tables S2 and S3 for details]. After accounting for the influence of perceived similarity [character: β = 0.50 [0.45, 0.55], SE = 0.03, *t*(796) = 19.0, *P* = 1 × 10^−66^; attitude: β = 0.44 [0.38, 0.51], SE = 0.03, *t*(796) = 14.1, *P* = 1 × 10^−40^], we found that the effect of perceived coherence [character: β = 0.35 [0.30, 0.40], SE = 0.03, *t*(796) = 12.8, *P* = 2 × 10^−34^; attitude: β = 0.35 [0.29, 0.41], SE = 0.03, *t*(796) = 11.1, *P* = 1 × 10^−26^] remained significant, as did the effect of character certainty [character: β = 0.08 [0.04, 0.13], SE = 0.02, *t*(796) = 3.57, *P* = 4 × 10^−4^]. By contrast, attitude certainty became nonsignificant [β = 0.05 [0.00, 0.10], SE = 0.03, *t*(796) = 1.82, *P* = 0.07].

The most noteworthy difference with respect to our previous studies is that the effect of perceived similarity remained significant after including perceived coherence and certainty as predictor variables (Fig. 5F). However, on the basis of the observation, replicated in studies 1 and 2, that coherence and certainty concurrently modulated both similarity and liking (Fig. 4), we studied whether this model also explained the variables collected in study 3. Consistent with this idea, we found that the confounding variable model, where perceived coherence and certainty increase both perceived similarity and liking, explained the data better than the previously considered mediation model (character: δAIC = 2.0; attitude: δAIC = 15.0; fig. S4). This provides further evidence suggesting that coherence and certainty are not mediators of the liking-by-similarity effect.

Last, we examined whether these variables could be moderators of the effect of similarity. A multivariate regression analysis including those effects suggested that the interaction between similarity and these variables is small and statistically nonsignificant. This absent interaction was observed in study 1 [coherence: β = −0.02 [−0.06, 0.02], SE = 0.02, *t*(2626) = −0.85, *P* = 0.39; certainty: β = 0.005 [−0.037, 0.046], SE = 0.021, *t*(2626) = 0.22, *P* = 0.83], study 2 [coherence: β = −0.01 [−0.09, 0.06], SE = 0.04, *t*(832) = −0.35, *P* = 0.73; certainty: β = 0.00 [−0.08, 0.08], SE = 0.04, *t*(832) = −0.01, *P* = 0.99], and also in study 3 [coherence: β = 0.00 [−0.04, 0.05], SE = 0.02, *t*(794) = 0.19, *P* = 0.85; certainty: β = 0.01 [−0.04, 0.06], SE = 0.02, *t*(794) = 0.43, *P* = 0.66]. This finding indicates that coherence and certainty are not moderators of the liking-by-similarity effect.

Summarizing, although study 3 was performed with a different methodology and sample from the ones used in studies 1 and 2, the data still show evidence that people are attracted to politically coherent and certain individuals. Moreover, this experimental study suggests the existence of a causal effect of the target’s coherence and certainty on interpersonal liking. Last, we observed that perceived coherence and certainty explain ratings of interpersonal attraction over and above the influence of perceived similarity.

## DISCUSSION

These results indicate that liking in the political domain may not be solely driven by homophily but by more complex notions of group affiliation that are reflected by two variables: coherence and certainty. The effect of the former quantity, i.e., coherence, indicates that people favor individuals who clearly share their same political orientation (*55*) and may suggest that they penalize individuals not taking sides (*56*). The modulation of liking by the latter variable, i.e., certainty, is in line with previous theory and evidence suggesting that confidence serves as a modulator of social influence (*57*, *58*). Our data also reveal that the liking-by-similarity effect (Fig. 1E) might be partially confounded by the effects of coherence and certainty (Fig. 4 and fig. S4), which modulate both variables. Furthermore, our findings suggest that symmetric rules might not fully explain interpersonal liking in the political domain (Fig. 2, G and J).

While we emphasize that our samples might not be representative of the general population (a caveat that applies to almost all psychological studies involving human participants), the claims presented in this work are supported by converging evidence from quantitative model comparison analyses (Table 1 and Figs. 3 and 4), the replication of our observational findings in a different language and political context (Fig. 3 and table S1), and an experimental study where we manipulated the variables of interest (Fig. 5). However, future research should explore whether and how these effects generalize to other settings, including participants from other populations.

From a theoretical perspective, this work has clear implications for studies that aim to model the dynamics of social systems. Most research in this growing field has worked under the reasonable assumption that individuals interact in social networks with varying degrees of homophily (*59*–*62*). Here, we show that liking ratings in the political domain are better explained by an asymmetric model. While our studies cannot directly speak about behavioral interactions and actual patterns of affiliation, the observed results suggest that like-attract-like rules might not be sufficient to explain feelings of attraction between individuals. This is also consistent with the finding that interaction rules based only on political similarity cannot produce complex collective phenomena, such as the polarization of political attitudes (*61*).

Previous research has shown that politically ambivalent individuals differ from people displaying clear opinions in both cognitive and behavioral aspects. For example, they are more tolerant to ambiguity (*63*), use more benevolent language (*64*), represent the world in a more complex and less clustered way (*65*), have more insight into the correctness of their choices (*66*), and promote the construction of consensus in polarized issues (*39*). Here, we add to this literature by showing that individuals with ambivalence or uncertainty in their political opinions are less likeable than people with coherent and confident views.

One limitation of this work is that the effects of coherence and certainty cannot be disentangled from the one given by participants’ political extremity (*11*, *17*, *19*). This is because our measurement instruments do not allow us to define two similar levels of coherence or certainty with different extremity, or vice versa. Therefore, our studies cannot establish whether the effects of coherence and certainty occur beyond the contribution of political extremity on liking.

Moreover, given that extreme individuals tend to be politically coherent and certain, the findings reported in this work seem to be at odds with previous research suggesting that people dislike strong partisans (*67*, *68*). However, as previously argued (*20*), people holding politically coherent opinions may not necessarily be strong partisans, as they simply could hold liberal or conservative views without being supporters of any specific political party. Alternatively, the incongruence between findings could stem from large methodological differences between this work and previous research reporting the phenomenon of partisan disdain. For example, some of these studies asked participants to rate whether they like individuals “who frequently talk about politics” without making any assumption about whether the individuals express opinions with high or low coherence (*67*). Future research should examine whether and how the negative effect of partisanship on liking interacts with the observation that coherence and certainty increase interpersonal attraction.

The effects of coherence and certainty on liking were strongly present in ratings of perceived intelligence (fig. S3). Therefore, one limitation of the results described in this work is that we cannot rule out whether the more coherent and confident individuals were indeed relatively more intelligent than ambivalent and uncertain participants. The data presented in study 3, which proceed from an experimental study where we manipulated the target’s opinions, suggest that this potential effect of actual intelligence does not fully explain the observed modulations by coherence and certainty.

To summarize, this research provides empirical evidence that political coherence and certainty predict interpersonal liking above and beyond the well-established effect of attitude similarity. While interaction rules based on individuals rewarding similarity have been attributed as the main causes of the existence of echo chambers in social media (*9*, *69*) and the political segregation of societies (*7*), this work suggests that such an assumption may oversimplify the complexity of political interactions. Future research should examine whether and how the asymmetric effects reported in this work relate to emergent phenomena such as the extremification of political opinions (*17*), the polarization of beliefs (*70*), and the disappearance of moderate views (*15*).

## MATERIALS AND METHODS

### Context

Study 1 took place on 24 October 2017 during a TEDx event in Buenos Aires, Argentina (http://tedxriodelaplata.org/). Study 2 took place on 6 April 2019 during another TEDx event in Porto, Portugal (https://tedxporto.com/). This was part of a program to perform experiments with live crowds (http://tedxriodelaplata.org/tedxperiments). Previous editions studied the use of a competition bias in a zero-sum fallacy game (*37*), the role of deliberation in the wisdom of crowds (*38*), and the factors that promote consensus in polarized moral issues (*39*).

### Participants

We collected data from 5727 participants in study 1 (57.3% female, aged means ± SD: 28.7 ± 10.8 years) and 877 participants in study 2 (62.0% female, aged 37.0 ± 11.4 years). Data were completely anonymous, and participants gave written informed consent. The experimental protocol was approved by the ethics committee of Centro de Educación Médica e Investigaciones Clínicas Norberto Quirno (Buenos Aires, Argentina), protocol 435, version 5.

### Materials

Research assistants handled one pen and one closed envelope containing an A4 paper folded on the long edge. The answer sheet had four pages. On page 1, participants were informed about their participant number and reported their age and gender. The three phases of the experiment were completed on pages 2, 3, and 4.

### Studies 1 and 2: Experimental procedure

The speakers (author J.N. in study 1 and authors D.A. and M.S. in study 2) announced that their section would involve a behavioral experiment. Participants were informed that their participation was completely voluntary and that they could simply withdraw their participation at any time.

### Phase 1: Individual opinions

Participants read five statements and were asked to report whether they agreed, disagreed, or were uncertain about their answer (Fig. 1B). In study 1, approximately half of the audience (52%) considered five political statements, while the remaining participants were presented with five hedonic statements. In study 2, all participants read five political statements. The order of presentation of the statements was different for different dyads. In both cases, participants were given 2 min to complete this section.

### Phase 2: Free discussion

In the second part of the experiment, participants freely discussed their opinions in dyads. First, they were instructed to find the other participant based on a numerical code presented on page 1. Answer sheets were distributed across the auditorium in such a way that dyads would be organized across consecutive rows. Participants were instructed to freely discuss the five statements for 5 min. They were explicitly told that there was no need to reach consensus or persuade the other participant and that they should only exchange opinions. To make sure that all opinions were interchanged during the discussion, we asked participants to write down on their own paper all the other person’s responses to the five statements.

### Phase 3: Interpersonal attraction scale

On the last page of the answer sheet, participants were given 2 min to complete five items using a five-point Likert scale (between “strongly disagree” and “strongly agree”). The five items were as follows: (i) She/he seemed nice; (ii) she/he seemed charming; (iii) she/he seemed intelligent; (iv) she/he seemed candid; (v) she/he was physically attractive. Participants were explicitly instructed to not discuss their answers to this scale and to reinsert the answer sheet in the envelope as soon as they completed the scale. In this page, participants could also indicate whether they knew the other participant from before the event. This allowed us to confirm that most interactions were between people who did not know each other (study 1: 88.0% and study 2: 95.4%).

### Selection of the statements

To select the statements used in study 1, we wrote a total of 28 statements and validated them in an online survey (*N* = 182) distributed to undergraduate students at Universidad Torcuato Di Tella. Each respondent to the survey considered 14 statements randomly selected from that list and indicated whether they agreed, disagreed, or did not know how to answer. They also indicated whether they believed that each statement was about politics using a 10-point Likert scale. We selected five political and five hedonic statements without a clear majoritarian opinion. The probability to agree to the selected statements was 43.7 ± 1.6%. We also confirmed that the statements that we refer to as “political” in study 1 were indeed perceived as more political than hedonic statements [8.7 ± 0.1 for political statements, 1.42 ± 0.06 for hedonic statements, two-sample *t* test, *t*(912) = 60.6, *P* < 1 × 10^−300^].

We implemented the same selection procedure for study 2. Respondents to the survey (*N* = 50) were recruited online through a mailing list of previous participants of TEDxPorto. Each individual completed a survey consisting of 20 statements. As in study 1, the five selected statements did not have a clear majoritarian answer. The probability to agree to the selected statements was 62.4 ± 3.1%.

### Selected statements

The five political statements used in study 1 (which was conducted in Buenos Aires, Argentina) were as follows: (a) High school students should be allowed to go on strike (“students”), (b) the government should mandate a transgender hiring quota for public servants (“trans quota”), (c) the government should subsidize the broadcasting of Argentine football matches (“football”), (d) national universities should start charging a fee to those who can afford it (“universities”), and (e) Argentina should sign bilateral trade agreements with the United States (“USA”). Three of them were framed to support a left-wing opinion (i.e., statements a, b, and c), whereas the remaining two supported a right-wing opinion (i.e., statements d and e). The five hedonic statements used in study 1 were as follows: (a) One should choose to adopt a dog over a cat (“pet”), (b) everyone should use a bidet if they have the possibility (“bidet”), (c) all good barbeques must have blood sausages (“BBQ”), (d) baked schnitzels taste better than fried schnitzels (“schnitzels”), and (e) it is better to go on holidays to the mountains rather than to the seaside (“holidays”).

The five statements used in study 2 (which was conducted in Porto, Portugal) were as follows: (a) The government should mandate a female hiring quota for public servants (“gender quota”), (b) everyone should use public transportation instead of personal cars (“transport”), (c) smoking should be forbidden in all public spaces (“smoking”), (d) drug consumption should not be completely decriminalized (“drugs”), and (e) universities should charge a fee to everyone (“universities Porto”). As in study 1, three of the statements were framed to support a left-wing opinion (i.e., statements a, b, and c), whereas the remaining two supported a right-wing opinion (i.e., statements d and e).

### Control study

To evaluate whether the selected statements were actually perceived as supporting a stereotypical left-wing or right-wing political opinion, we performed a control study. Participants were undergraduate students at Universidad Torcuato Di Tella recruited online (*N* = 35, 21 females, aged 18 to 24). The study had two parts. In the first part, framed as a two-alternative forced choice, participants indicated whether each of the five selected statements reflected a left-wing or a right-wing opinion. In the second part, participants were told to imagine the responses given by six individuals showing different responses to these statements. These profiles were presented in random order, and participants were asked to infer the political position of the target individuals by using a six-point Likert scale (with its two extremes labeled as “left-wing extreme” and “right-wing extreme”).

### Exclusion criteria

At the end of each event, we collected the papers as the participants exited the auditorium. Data entry research assistants digitalized the data using a computer. We discarded data from participants who had not completed all three phases of the experiment and from dyads who reported to have known each other from before the event. Overall, we report results from *N* = 5038 participants in study 1 (2911 females, mean age ± SD: 28.9 ± 10.8 years, 52.2% who read political statements) and *N* = 838 participants in study 2 (523 females, aged 36.9 ± 11.3 years, all considering political statements).

### Measuring the variability of opinions within the crowd

One possible concern about the experimental procedure is that dyads were formed by physical proximity. Because participants sitting in neighboring seats typically know each other, they could also potentially share similar opinions. To reduce the chances of this happening, we asked people to organize in groups across different rows, which led to a large fraction of interactions between people who did not know each other from before the event (study 1: 88.0% and study 2: 95.4%). To test whether the assignment of dyads across consecutive rows was random relative to the opinions provided by the crowd, we performed a random permutation analysis. We created 10,000 synthetic datasets by randomly shuffling the matching between dyads in the crowd. We then computed the similarity of opinions for each dyad and each simulation. From these 10,000 surrogates, we chose the one with median similarity and compared it to our empirical observations. We found that the median synthetic and real data had overlapping distributions [real data: mean = 2.0, SD = 1.2, median = 2, interquartile range (IQR) = 2; synthetic data: mean = 1.9, SD = 1.2, median = 2, IQR = 2] with a negligible effect size (Cohen’s *d* = 0.15). This suggests that the matching between individuals was similar to a random procedure.

### PCA of individual opinions

To test for possible correlations between the responses to the statements, we performed a PCA. In study 1, we found that the first principal component explained 37.0% of the variance of the political opinions and 27.3% of the variance of hedonic responses. The second principal component explained a small fraction of the variance, which was, in both cases, close to chance level (19.1% for politics and 21.3% for hedonics; chance level, 20%). To measure the significance level of these fractions, we performed a random permutation analysis. This was done by randomly shuffling the matching between responses to different statements and repeating this procedure in 10,000 simulations. We observed that only the first principal component explained a significant proportion of the variance, both for political (*P* < 1 × 10^−4^ for the first principal component and *P* > 0.9 for the second component) and hedonic (*P* < 1 × 10^−4^ and *P* = 0.15) opinions. A similar pattern was observed in study 2 (first principal component: 27.0%, *P* < 1 × 10^−4^; second principal component: 22.6%, *P* = 0.5). Hence, we projected the agree/disagree opinions to their first principal component, which (in the political domain) was equivalent to coding them as stereotypical left/right-wing answers.

### Variables characterizing individuals

We coded left-wing answers (L) with a value of −1, right-wing answers (R) with a value of +1, and the opt-out option (“?”) with a value of 0. We then defined the political position π^(*j*)^ of participant *j* as

$$\pi^{\left(j\right)}=\sum_{i=1}^{n}x_{i}^{\left(j\right)}$$ (1)

where $x_{i}^{\left(j\right)}$ is the *i*th answer of participant *j*, and *n* = 5 is the number of statements. This quantity could take any integer value between −5 and +5 (with more positive values indicating a position further to the right) and reflects the signed difference between the number of right-wing and left-wing opinions.

Similarly, we define the political orientation θ^(*j*)^ of participant *j* as the sign of his/her political position

$$\theta^{\left(j\right)}=\text{sign}(\pi^{\left(j\right)})$$ (2)

If a participant provided more left-wing than right-wing answers, we said that he/she was oriented to the left, and we coded that individual with a value of −1. Conversely, if an individual gave more right-wing than left-wing responses, then his/her political orientation had a value of +1.

We also defined the internal coherence κ^(*j*)^ of participant *j* as the absolute value of his/her political position

$$\kappa^{\left(j\right)}=|\pi^{\left(j\right)}|$$ (3)

and his/her certainty ξ^(*j*)^ as the number of responses where the participant did not use the opt-out option

$$\xi^{\left(j\right)}=\sum_{i=1}^{n}|x_{i}^{\left(j\right)}|$$ (4)

### Variables characterizing dyadic interactions

All the variables in Eqs. 1 to 4 denote features of the responses provided by participants and not the interaction between them. To define the variables that characterize the interaction between participants *j* and *k*, we used two supra-indexes: one denoting the participant who rated (i.e., *j*, the first person) and one denoting the participant that was being rated (i.e., *k*, the second person).

Following a vast literature suggesting that liking is primarily modulated by the number of shared opinions (*36*), we defined the similarity *S*^(*j*, *k*)^ between two individuals as

$$S^{\left(j,k\right)}=\sum_{i=1}^{n}H(x_{i}^{\left(j\right)}\;x_{i}^{\left(k\right)})$$ (5)

where *H* is the Heaviside step function, which takes a value of +1 for positive arguments and is 0 otherwise. This definition ensures that similarity only counts well-defined shared opinions (i.e., if both participants are uncertain about a given statement, that does not count as a shared opinion). However, our main results do not depend on this specific definition of similarity (see the “Other definitions of similarity” section below). Notice that *S* is a pseudo-metric given that it is positive [*S*^(*j*, *k*)^ > 0 for all values of *j* and *k*] and symmetric [*S*^(*j*, *k*)^ = *S*^(*k*, *j*)^] but does not satisfy the identity of indiscernibles [*S*^(*j*, *k*)^ = 0 does not necessarily imply that $x_{i}^{\left(j\right)}=x_{i}^{\left(k\right)}$for all values of *i*].

We defined the ingroup coherence *C*^(*j*, *k*)^ in a dyadic interaction as

$$C^{\left(j,k\right)}=\theta^{\left(j\right)}\;\pi^{\left(k\right)}$$ (6)

This quantity takes positive values for interactions between individuals with the same political orientation [i.e., θ^(*j*)^ = θ^(*k*)^], negative values for interactions between people with opposite political orientations [i.e., θ^(*j*)^ = − θ^(*k*)^], and 0 otherwise. Moreover, this variable increases in absolute value as the second person (i.e., the individual being rated) becomes more internally coherent [i.e., κ^(*j*)^ becomes larger]. Overall, this definition suggests that people may feel most (least) attracted to ingroups (outgroups) who are clearly aligned with their political orientation. This quantity is asymmetric [*C*^(*j*, *k*)^ ≠ *C*^(*k*, *j*)^] and predicts that incoherent individuals should like coherent ingroups more than vice versa.

From a mathematical viewpoint, ingroup coherence could be regarded as the interaction between the participant’s political orientation [θ^(*j*)^] and the target’s political position [π^(*k*)^]. Hence, one reasonable question is whether the effect of coherence remains significant after including the main effects given by θ^(*j*)^ and π^(*k*)^. We observed that liking continued to be modulated by *C*^(*j*, *k*)^ [β = 0.13 [0.09, 0.17], SE = 0.02, *t*(2628) = 6.1, *P* = 1 × 10^−9^] while controlling for the main effects of θ^(*j*)^ [β = 0.07 [0.03, 0.11], SE = 0.02, *t*(2628) = 3.5, *P* = 0.0005] and π^(*k*)^ [β = −0.03 [−0.06, 0.01], SE = 0.02, *t*(2628) = −1.3, *P* = 0.20].

### Other definitions of similarity

To further evaluate the validity of our main results, we tested whether they were robust to other mathematical definitions of similarity. For example, the definition in Eq. 5 only considers a match between two opinions if neither of them used the opt-out options (i.e., agreeing to be uncertain was not counted as sharing the same opinion). We found that if those cases counted toward similarity, then the restricted model would still provide poorer fits to the data compared to the proposed model (δAIC = 18.8). In addition, if we considered that equal opinions increased similarity, opposite opinions decreased similarity, and combinations including uncertain responses did not change similarity, then the univariate model would still perform worse than the one based on coherence and certainty (δAIC = 18.0). Last, if a combination of an uncertain response with a well-defined one counted half as much as a matching between well-defined responses, then this definition of similarity would also lead to worse fits (δAIC = 21.6).

### Linear mixed-effects regressions and model comparisons

We modeled liking ratings using linear mixed-effects regressions where we added random terms for each dyad. The variables of interest (i.e., similarity, coherence, and certainty) were standardized and included as fixed effects. As our proposed model is an extension of the univariate liking-by-similarity model, we performed likelihood-ratio tests to evaluate whether the data were significantly better explained by the multivariate model. In addition, to compare the goodness of the fits, we used a cross-validation approach and measured the out-of-sample accuracy of both models. To perform this analysis, we used a random 50/50 split of the data to define the training and testing sets. We fitted both models to the training set and then measured the MSE and log-likelihood of the testing set. We repeated this procedure 10,000 times and compared the difference in out-of-sample MSE and log-likelihood between the two models.

### Structural equation models

We fitted different structural equation models where we included different links between similarity, coherence, certainty, and liking (Fig. 4 and fig. S4). To do this, we used the lavaan R package (*71*) and reported coefficient estimates, SEs, and the *P* values for testing the null hypothesis that each parameter is equal to zero. Model comparisons were done using the AIC. Causal mediation analysis was performed using the mediation R package (*72*) and bootstrapping tests where we ran 1000 iterations. We report coefficient estimates, 95% confidence intervals, and the *P* values for testing the null hypothesis that the effects are equal to zero.

### Study 3: Experimental manipulation of coherence and certainty

We performed a preregistered study where participants read the opinions of two putative Twitter users then rated whether they believed that the person expressing those opinions was similar to themselves, their political coherence, and their political certainty. Last, they completed a standard interpersonal attraction scale.

Each target expressed six opinions about different political issues in the United States: immigration, global warming, police brutality, same-sex marriage, gun control, and COVID-19 vaccines. Each profile had a name (James/Robert for male targets; Patricia/Linda for female targets) and was accompanied by an artificial face created through the website of Generated Media Inc. (https://generated.photos/) (*73*). Tweets were framed in a way in which they could either express a liberal opinion, an ambiguous opinion, or a conservative one. The content of these 18 tweets is displayed in table S4. The gender of the two profiles and their order of presentation were randomly chosen for each participant.

After reading all tweets of a given user, participants provided ratings of perceived attitude similarity, coherence, and certainty. We asked them to report, “To what extent do you feel that this person holds (similar political views to yours/coherent political views/confident political views)?” We also asked participants to indicate whether they believed that the target was similar, coherent, and certain in character. Specifically, they were asked, “To what extent do you feel that this person is (similar in character to you/coherent in character/confident in character)?” In both cases, participants provided their answers using a five-point Likert scale (1: “not similar/coherent/confident at all” to 5: “highly similar/coherent/confident”).

We then measured interpersonal liking through six items (Cronbach’s α = 0.95): (1) I think she/he could be a friend of mine; (2) she/he would perfectly fit into my circle of friends; (3) if I wanted to get things done, I could probably depend on him/her; (4) how much do you think you would like this person?; (5) how much would you want to work with this person?; (6) how warm do you feel about this person? Each item was completed using a five-point Likert scale (items 1 to 3: from “I completely disagree” to “I completely agree”; items 4 to 6: from “not at all” to “very much”). Our dependent variable (liking) was computed as the mean rating across the six items.

Participants were pseudo-randomly assigned to two between-subjects conditions. In condition 1 (*n* = 200), they rated two targets who had different degrees of coherence in their attitudes. For liberal participants, one target had six liberal opinions (high coherence), and the other target had four liberal opinions and two conservative opinions (low coherence). Conservative participants assigned to this condition rated one target with six conservative opinions (high coherence) and one target with four conservative opinions and two liberal opinions (low coherence). In condition 2 (*n* = 200), participants rated two targets who had different degrees of certainty in their opinions. For liberal participants, one target had five liberal opinions and one conservative opinion (high certainty), and the other target had four liberal opinions and two uncertain opinions (low certainty). Conservative participants assigned to this condition rated one profile with five conservative opinions and one liberal opinion (high certainty), and the other target had four conservative opinions and two uncertain opinions (low certainty).

We performed four planned analyses. Analysis #1 consisted of comparing liking ratings in the high-coherence versus the low-coherence condition. Analysis #2 was set to compare liking across the high-certainty and low-certainty conditions. In both cases, we performed a Wilcoxon signed-rank test for equal medians using a 5% significance level. Analyses #3 and #4 consisted of fitting linear mixed-effects models on liking with three predictor variables: perceived similarity, perceived coherence, and perceived certainty. While analysis #3 was done with perceived attitude similarity/coherence/certainty, analysis #4 was performed with perceived character similarity/coherence/certainty. As preregistered, the regression clustered values on participants by fitting random intercepts for each individual. We tested for significance using a 5% significance level.

We collected data from *N* = 400 participants obtained through Prolific (aged 26.5 ± 8.5 years, 50% female/male, and 50% self-reported as liberal/conservative). Sample size was estimated through a power analysis based on data collected in a pilot experiment with *N* = 40 individuals recruited through the same platform and who did not participate in study 3. We performed a Monte Carlo Power Analysis procedure, where we constructed synthetic datasets by resampling the pilot data with replacement. For a given sample size, we ran 1000 simulations and performed analyses #1 to #4 on each iteration. Power was estimated as the fraction of times where we rejected the null hypotheses on each individual test. On the basis of this procedure, we estimated a power of more than 86% for all analyses (analysis #1: 99.9%; analysis #2: 99.5%; analysis #3: 86.8%; analysis #4: 99.4%). This power analysis is available at the Open Science Framework (https://osf.io/ayp4w/). The hypotheses of this study, its experimental design, and all planned statistical analyses were preregistered at https://aspredicted.org/q7kr6.pdf.
