# Supplementary material for: Political coherence and certainty as drivers of interpersonal liking over and above similarity
Source: Sci Adv. 2022 Feb 9;8(6):eabk1909. doi: 10.1126/sciadv.abk1909 (PMC8827732; doi:10.1126/sciadv.abk1909)
Supplement: Supplementary file 1 — Figs. S1 to S4 Tables S1 to S4 [file sciadv.abk1909_sm.pdf]

Science Advances
AAAS

Supplementary Materials for

Political coherence and certainty as drivers of interpersonal liking over and above similarity

Federico Zimmerman, Gerry Garbulsky, Dan Ariely, Mariano Sigman, Joaquin Navajas*

*Corresponding author. Email: joaquin.navajas@utdt.edu

Published 9 February 2022, *Sci. Adv.* **8**, eabk1909 (2022)
DOI: 10.1126/sciadv.abk1909

This PDF file includes:

Figs. S1 to S4
Tables S1 to S4

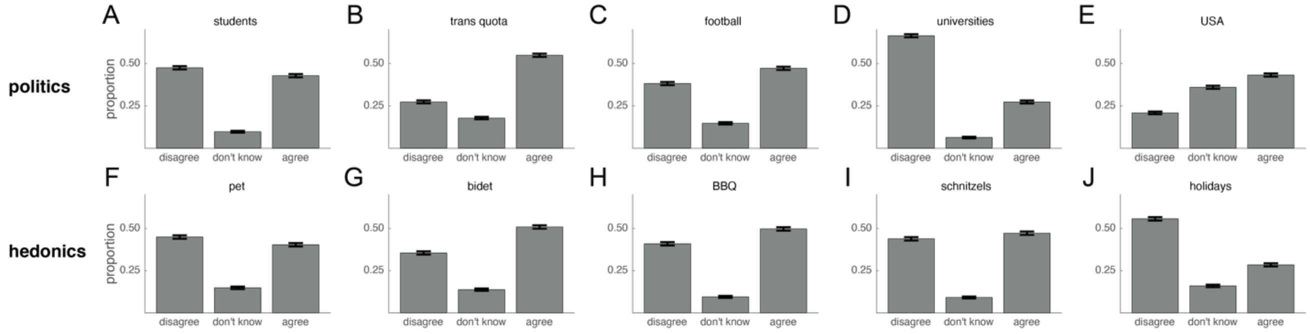

Fig. S1. Normalized histograms for individual opinions of phase 1. The height of the bars shows the proportion of participants that selected the 'disagree' (left bar), 'don't know' (middle bar), and 'agree' (right bar) option for each of the 10 statements used in Study 1. Error bars depict standard error of proportion. Upper panels **(A-E)** show political statements and lower panels **(F-J)** show hedonic statements. For a full description of the selection procedure of the statements, see Materials and Methods.

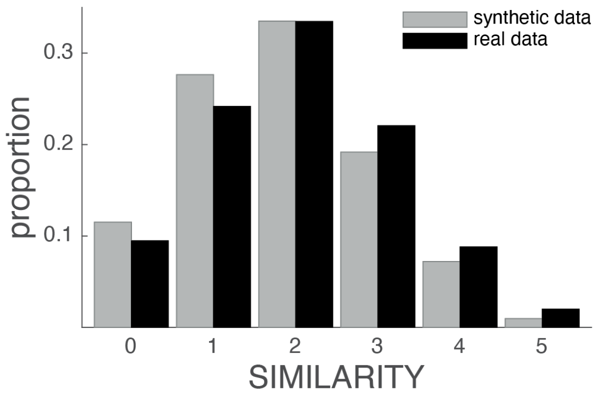

Fig. S2. Measuring the variability of opinions within the crowd. Black bars show the histogram of similarity, defined as the number of shared opinions, observed in Study 1. To evaluate how this distribution compares to a random procedure, we created 10,000 surrogates where we randomly reorganized dyads in the crowd. The gray bars show the histogram of similarity for the most representative simulation, defined as the one with median similarity across simulations.

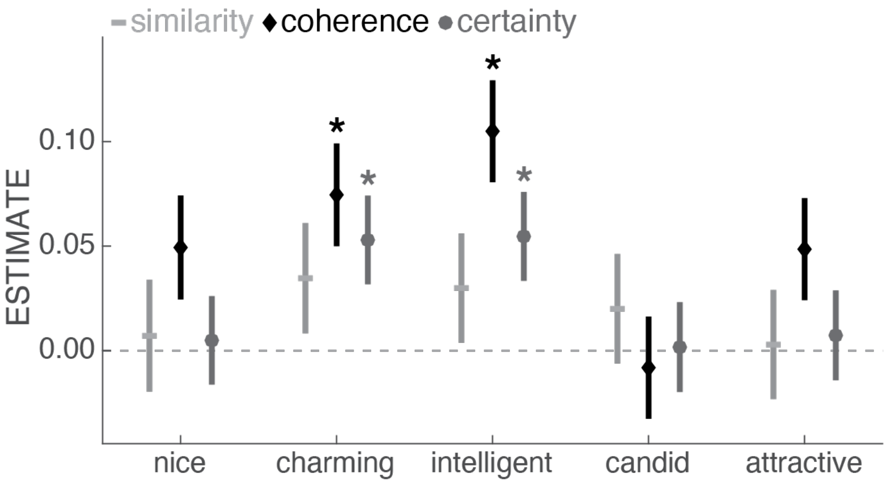

Fig. S3. Multivariate mixed model for each individual item in the interpersonal attraction scale. Considering each of the individual items of the interpersonal attraction scale separately, we fitted five different multivariate mixed models. We show the estimate values of each variable for the five different items. Similarity estimates are shown in light gray lines, coherence estimates are shown in black diamonds and certainty estimates in dark gray circles. Vertical lines show SEM and asterisks show significant effects (in all cases, $p<.01$). We observed that the main results were driven by the items where participants rated the charm and intelligence of their partner.

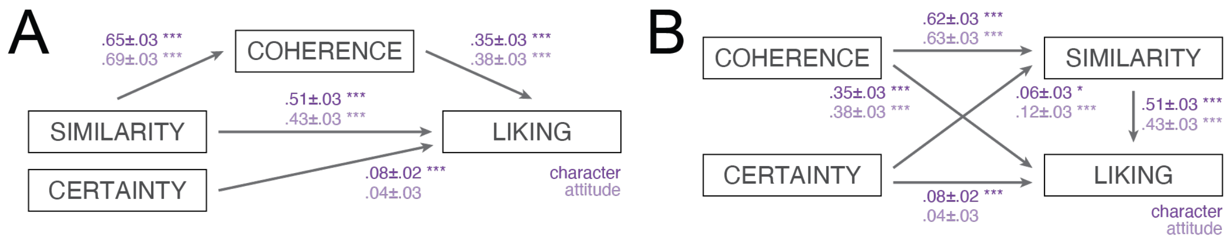

Fig. S4. Interplay between perceived similarity, coherence, certainty, and liking. Using the data collected in Study 3 (N=400), we repeated the analyses performed in **Fig. 4**. (**A**) Mediation (MED) model. We fitted a model where the effect of similarity on liking is mediated by coherence while certainty directly predicts liking. This model provided better fits than a model where certainty is the mediator. (**B**). Confounding variable (CV) model. We fitted another model where similarity and liking are both driven by coherence and certainty. This account would suggest that the liking-by-similarity effect is confounded by the influence of coherence and certainty on both variables. In both panels, we display the best-fitting coefficients ± S.E. and asterisks indicate the significance level of each estimate (*: p<.05, **: p<.01, ***: p<.001). Dark purple: perceived character similarity / coherence / certainty; light purple: perceived attitude similarity / coherence / certainty. Our observations are more likely under the CV model than under the MED model.

Study 2	Restricted Model			Full Model		
	β	t	p	β	t	p
SIMILARITY	.101±.036	2.83	.005	.055±.041	1.33	.18
COHERENCE	-			.041±.039	1.04	.30
CERTAINTY	-			.096±.036	2.65	.008
Log-likelihood	-1181			-1178		
AIC	2371			2367		
Likelihood ratio test	$\chi^2(2)=7.5$, $p=.02$					

Table S1. Comparison between the full multivariate mixed model and the restricted model for Study 2. Columns show the standardized coefficient estimates ± SEM, t-values, and p-values of each predictor. For each model, rows depict similarity (Equation [5]), coherence (Equation [7]), certainty (Equation [4]), log-likelihood and the Akaike information criterion (AIC). Finally, we tested whether the full model provides a significant better fit of the observed data by performing a likelihood-ratio test and we were able to replicate our findings.

Study 3 - Character	**Restricted Model**			**Full Model**		
	β	t	p	β	t	p
SIMILARITY	.77±.02	34.0	$4x10^{-157}$	.50±.03	19.0	$1x10^{-66}$
COHERENCE	-			.35±.03	12.8	$2x10^{-34}$
CERTAINTY	-			.08±.02	3.57	$4x10^{-4}$
Log-likelihood	-766			-661		
AIC	1539			1334		
Likelihood ratio test	$\chi^2(2)=209$, $p<1\times10^{-300}$					

Table S2. Comparison between the full multivariate mixed model and the restricted model for Study 3 - Character. Columns show the standardized coefficient estimates ± SEM, t-values, and p-values of each predictor. For each model, rows depict perceived character similarity / coherence / certainty, log-likelihood and the Akaike information criterion (AIC). Finally, we tested whether the full model provides a significant better fit of the observed data by performing a likelihood-ratio test and we were able to replicate our findings.

Study 3 - Attitude	**Restricted Model**			**Full Model**		
	β	t	p	β	t	p
SIMILARITY	.72±.02	30.5	$3x10^{-136}$	.44±.03	14.1	$1x10^{-40}$
COHERENCE	-			.35±.03	11.1	$1x10^{-26}$
CERTAINTY	-			.05±.03	1.82	.07
Log-likelihood	-819			-749		
AIC	1645			1509		
Likelihood ratio test	$\chi^2(2)=140$, $p<1\times10^{-300}$					

Table S3. Comparison between the full multivariate mixed model and the restricted model for Study 3 - Attitude. Columns show the standardized coefficient estimates ± SEM, t-values, and p-values of each predictor. For each model, rows depict perceived attitude similarity / coherence / certainty, log-likelihood and the Akaike information criterion (AIC). Finally, we tested whether the full model provides a significant better fit of the observed data by performing a likelihood-ratio test and we were able to replicate our findings.

Issue	Liberal	Ambiguous	Conservative
Immigration	Immigration is good for the US. #AllowImmigration #AmericanDream	I still haven't made up my mind about whether immigration is good or bad. #StopImmigration #AllowImmigration	Immigration is bad for the US. #StopImmigration #AmericaforAmericans
Global warming	There is no Planet B. Global warming should be taken seriously! #GlobalWarming #ClimateChange	Global warming may or may not be happening. There is too much uncertainty! #GlobalWarming #GlobalCooling	Global warming is not a thing. Stop scaring people! #GlobalCooling #ClimateChangeHoax
Police brutality	Police brutality against black people must stop. At what point do we say enough is enough? #BlackLivesMatter #DefundThePolice	Are black people treated differently by the police? I'm not convinced this is really an issue. #BlackLivesMatter #AllLivesMatter	Our police officers deserve better protection and recognition from politicians and from this country as a whole. #AllLivesMatter #FundThePolice
Same-sex marriage	Same-sex marriage is a basic human right. It must be legal! #SameSexMarriage #GayPride	Same-sex marriage is a complex issue. I'm not sure whether it should be prohibited or allowed. #SameSexMarriage #1Man1Woman	"Marriage" is between a man and a woman. Same-sex marriage must always be ilegal. #1Man1Woman #GayShame
Gun control	People are dying. We must stop mass shootings now. #GunControlNow #StopGunViolence	I understand mass shootings should be stopped but I'm not sure gun control is the way forward. #SecondAmendment #GunControl	The rights of every American to keep and bear arms must be protected. #SecondAmendment #NoGunControl
COVID-19 vaccines	Double jabbed. I am now part of the solution to end the pandemic. #VaccinesWork #GetVaccinated	COVID-19 vaccines may be good or bad. We still don't know yet! #VaccinesKill #VaccinesWork	Don't be lab rats to pharma companies. Vaccines are evil and dangerous! #VaccinesKill #DontGetVaccinated

Table S4. Opinions displayed by the profiles rated in Study 3. Each row displays a different issue and columns show a liberal (first column), and ambiguous (second column), or a conservative (third column) opinion for each topic. Opinions were displayed in tweets as in **Fig. 5.** All materials used in this study are available at the Open Science Framework (https://osf.io/ayp4w/).
